# Atlantoaxial rotatory fixation in childhood: a staged management strategy incorporating manipulation under anaesthesia

**DOI:** 10.1007/s00381-020-04727-y

**Published:** 2020-07-13

**Authors:** Ciaran Scott Hill, Anouk Borg, Muhammad Zubair Tahir, Dominic Nolan Paul Thompson

**Affiliations:** Department of Neurosurgery, Great Ormond Street Hospital for Children, London, UK

**Keywords:** Atlantoaxial rotatory fixation, Subluxation, Torticollis, Paediatric, Halo-body orthosis

## Abstract

**Aims:**

The aims were to evaluate the safety of manipulation under anaesthesia (MUA) for atlantoaxial rotatory fixation (AARF) and the relative efficacy of rigid collar vs halo-body orthosis (HBO) in avoiding relapse and the need for open surgery.

**Methods:**

Cases of CT-verified AARF treated by MUA were identified from a neurosurgical operative database. Demographic details, time to presentation and aetiology of AARF were ascertained through case note review. Cases were divided according to method of immobilisation after successful reduction, either rigid collar (group 1) or HBO (group 2). The primary outcome measure was relapse requiring open surgical arthrodesis.

**Results:**

Thirty-three patients (2.2–12.7 years) satisfied inclusion criteria. Time to presentation varied from 1 day to 18 months. There were 19 patients in group 1 and 14 in group 2. There were no adverse events associated with MUA. 9/19 (47%) patients in group 1 resolved without need for further treatment compared with 10/14 (71%) in group 2 (*p* = 0.15). Of the 10 patients who failed group 1 treatment, four resolved after HBO. A total of ten patients (30%) failed treatment and required open surgery.

**Conclusions:**

MUA is a safe procedure for AARF where initial conservative measures have failed. MUA followed by immobilisation avoids the need for open surgery in over two thirds of cases. Immobilisation by cervical collar appears equally effective to HBO as an initial management, and so a step-wise approach may be reasonable. Delayed presentation may be a risk factor for relapse and need for open surgery.

## Background

**Fig. 1:**
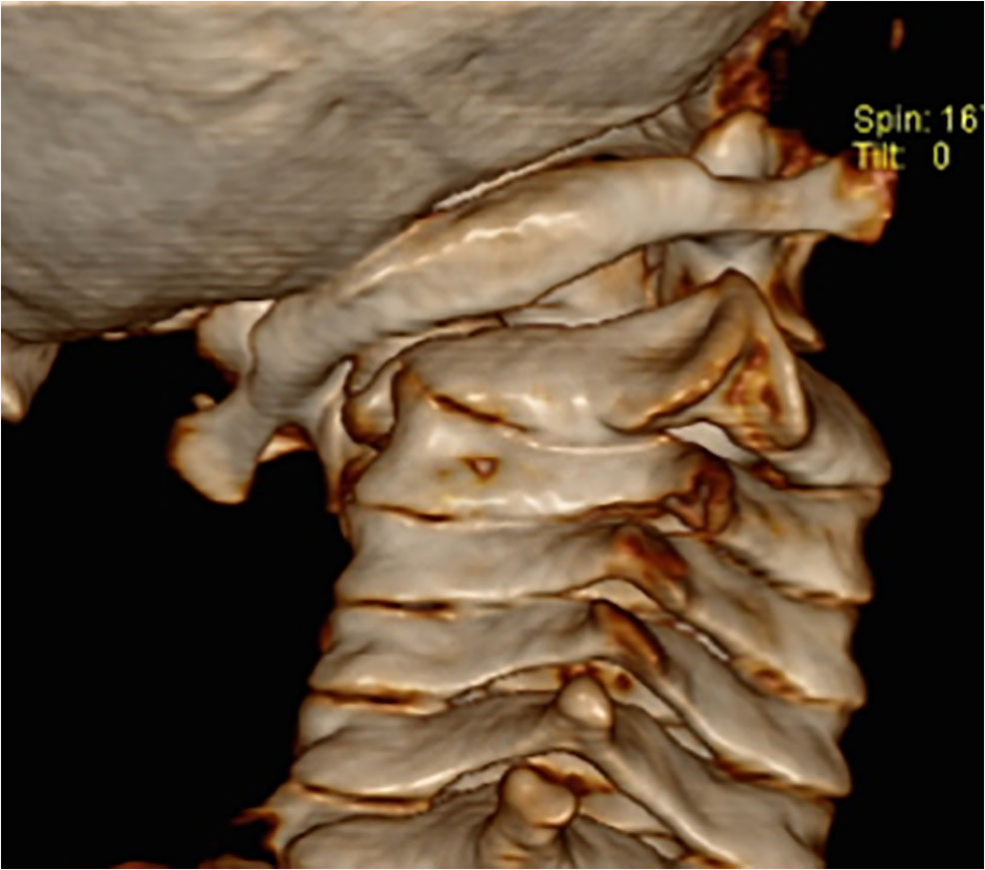
Rotational deformity of C1-C2 complex**.** *Computerised tomographic 3D reconstruction of the craniocervical junction demonstrating rotational deformity of C1 in relation to C2*

Atlantoaxial rotatory fixation (AARF) is an acquired fixed rotation abnormality of the C1 vertebra on C2 that impedes turning of the neck and typically manifests with painful torticollis. It is primarily a condition of childhood although adolescent and adult cases are seen [1–3]. Clinical diagnosis of AARF is suspected on the basis of a fixed rotational deformity of the neck, often with the typical “Cock-Robin” position created by cervical lateral flexion, forward flexion and rotation [1, 4, 5]. Various criteria for radiological diagnosis have been offered but, as a minimum, confirmation of the diagnosis requires dynamic CT in left and right rotation demonstrating rotatory deformity of C1 with respect to C2 which fails to correct on attempted contralateral movement (Fig. 1) [4, 6, 7]. It is important to differentiate from simple spasmodic torticollis due isolated spasm of the sternocleidomastoid muscle in order to avoid misclassification and overtreatment [8, 9].

Instability and neurological injury are rare, but delayed investigation and treatment may compromise the prospects for complete recovery [10–13]. When left untreated, the atlantoaxial joint is at risk of fusion in the abnormal position leaving the child’s neck fixed in torticollis. In addition to cosmetic effects, this can result in functional disability, and predispose to chronic pain and compensatory deformity in the subaxial spine, as well as facial asymmetry [10, 11, 13].

Management strategies for AARF are poorly defined both in the primary care setting and in specialised centres. Incorrect and delayed diagnosis are commonplace [8, 13, 14]. The literature confirms that early and effective closed treatment of AARF can result in excellent outcomes in the majority of cases; however, early relapse is well recognised and may necessitate open reduction of C1-C2, often with arthrodesis [15–17]. The use of traction is commonplace but can be impractical in children and requires prolonged hospital stay.

## Aims

The aims of this study were to audit our treatment outcomes for AARF in children managed by initial closed reduction by MUA followed by immobilisation. In particular, the aim is to evaluate the relative efficacy of cervical collar and halo-body orthosis (HBO) as means of post-reduction immobilisation. The primary outcome measure was failure of conservative treatment requiring open surgery.

## Methods

Cases of AARF treated by MUA were identified from the Great Ormond Street Hospital paediatric neurosurgery departmental database. A retrospective case note and radiology review was performed. Diagnosis of AARF was made based on a clinical presentation of new onset painful torticollis and confirmed with a fine cut dynamic CT of the cervical spine with bone windowing. MRI was not routinely performed as the incidence of spinal cord injury or compromise is small in this condition and the prognostic value of evaluating soft tissue/ligamentous injury is currently unproven.

The underlying cause, time to presentation and neurosurgical management pathway was described for each of the patients. Patients were divided into two groups according to the method of post-MUA immobilisation: rigid cervical collar (group 1) and HBO (group 2).

In the absence of a clear evidence base for the most appropriate means of post-reduction immobilisation, the options of hard collar vs HBO were discussed with parents prior to treatment. The final decision was based on the consensus view of the parents and treating surgeon rather than specific radiological features or ease of reduction.

## Inclusion and exclusion criteria

All the cases in these series had failed initial conservative treatment, which included combinations of analgesia, muscle relaxants and cervical collars for up to 2 weeks. Other treatment modalities that have been described in the literature such as sternocleidomastoid release and botulinum toxin injection were not used in any patients**.** Cases of torticollis secondary to fracture, tumours of the craniovertebral region and congenital malformations such as segmentation anomalies were excluded.

## Treatment paradigm

All children underwent manipulation and attempted reduction under general anaesthesia combined with fluoroscopic guidance. Intraoperative neurophysiological monitoring was not routinely used since neurological compromise is exceedingly rare in AARF. Anaesthesia was maintained with a supraglottic airway. Once under anaesthetic, gentle traction was applied, followed by rotation, counter to the direction of subluxation. Typically, the axis of rotation occurs about the contralateral atlantoaxial joint that is used as a pivot point during relocation. Following fluoroscopic confirmation of reduction, two alternative modes of initial immobilisation have been used for this group of patients, either a hard cervical collar (Miami J) or HBO body orthosis. Treatment success was defined as clinical resolution of the torticollis together with CT evidence of satisfactory restoration of alignment of the C1-C2 complex. Treatment failure was defined as relapse of AARF requiring open surgery and arthrodesis.

## Statistics

Statistical analysis was carried out using Stata version 16.0 and GraphPad Prism version 8.0. The Fisher exact test was used to on contingency tables data to determine statistical significance, defined as p < 0.05.

## Results

Between January 2003 and January 2020 data was available for 33 patients treated by MUA for acquired AARF at our unit. The mean age of patients at presentation was 8.4 years (range 2.2 to 12.7 years). There was a male to female ratio of 39:61. The duration of symptoms prior to presentation ranged from 1 day to 18 months. The mean time from onset to presentation was 4.7 months with a median of 2 months (interquartile range 7–12 months).

A traumatic event prior to the onset of AARF was documented in 14 cases (42%). Four cases (12%) had a clear history of a preceding nasopharyngeal infection (Grisel’s syndrome), and 2 cases (6%) occurred following mastoidectomy for chronic otitis media. Other causes are listed in Table 1.

**Table 1:** Causes or contributory conditions related to AARF in our series (*n* = 33)

Cause	Number (%)
Trauma	14 (42%)
Idiopathic	11 (33%)
Grisel’s syndrome	4 (12%)
Post-mastoidectomy	2 (6%)
Seizure	1 (3%)
Ehlers-Danlos syndrome	1 (3%)

Of the 33 patients, 19 were initially managed in a hard cervical collar (group 1) and 14 using HBO (group 2). There were no patients who received internal fixation as a primary treatment. The intention was to immobilise for a minimum of 6 weeks as this was considered and appropriate time to allow for resolution of muscle spasm and recovery of soft tissue/ligamentous injury. However, patient compliance meant this was not always achieved and duration of treatment ranged from 2 to 6 weeks for a hard collar and 6–12 weeks for HBO.

There were no significant differences in age, gender, aetiology or time to presentation between the two groups (Table 2).

**Table 2:** Demographic of the two treatment groups

	Group 1	Group 2	
Total number	19	14	
Age (years)			*p* = 0.5 (OR 0.76, 95% CI 0.09–5.01)
< 7 > 7	5 14	3 11	
Gender			*p* = 0.07 (OR 3.7, 95% CI 0.69–20.95)
Male	5	8	
Female	14	6	
Aetiology			*p* = 0.62 (OR 0.97, 95%CI 0.19–4.92)
Traumatic	8	6	
Other	11	8	
Time to presentation			*p* = 0.15 (OR 0.29, 95% CI 0.03–2.01)
< 1 month	7	2	
> 1 month	12	12	

### Group 1

**Fig. 2:**
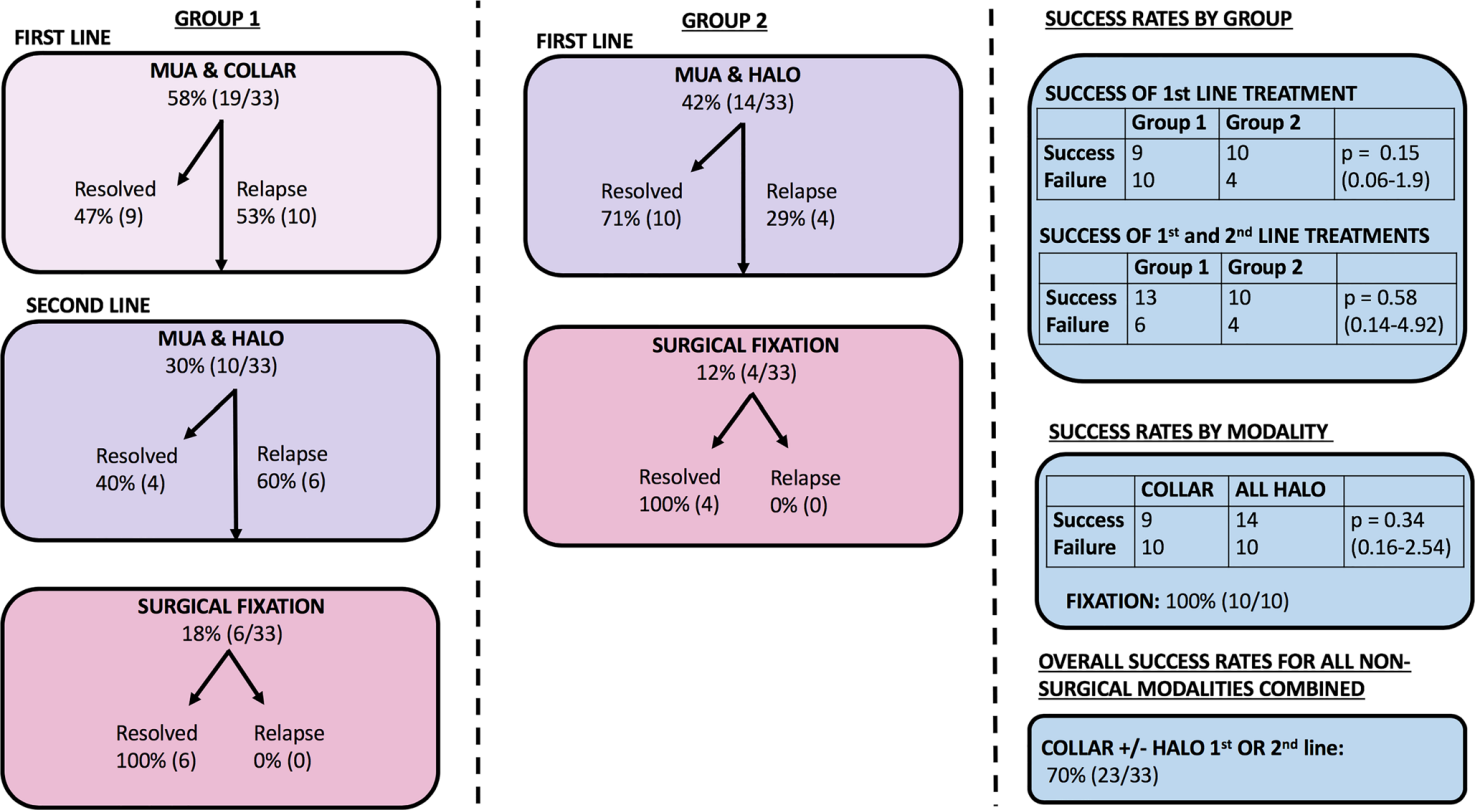
AARF outcomes. *AARF outcomes organised by grouped treatment strategy*

Forty-seven percent of patients (9/19) were successfully managed by closed reduction and cervical collar alone. The 10 patients who failed this initial treatment were treated by repeat closed reduction and immobilisation in a HBO; 4 resolved with this treatment and required no further intervention. The remaining 6 patients relapsed and went on to require reduction open surgery and arthrodesis. The conversion rate to surgery in group 1 was 6/19 (32%) (Fig. 2).

### Group 2

Ten of the 14 (71%) patients managed by initial closed reduction and HBO resolved and required no further treatment. The remaining 4 patients were successfully treated by open surgery and arthrodesis. Thus, the conversion rate to surgery in group 2 was 29% (Fig. 2).

Surgical fusion was avoided in 23/33 (70%) of the children. There was a trend toward greater success of HBO compared with cervical collar but this did not reach statistical significance.

The determinants of treatment success or failure have not yet been clearly defined in the literature, and a robust prognostication tool is notably absent. Based on clinical experience, we predefined four potential determinants of outcome and hypothesised without prejudice that they may determine rates of treatment failure. The effect of the following factors on treatment outcome was examined: time to presentation, aetiology of injury, age and gender. These were substratified by treatment group and analysed as shown in Table 3.

**Table 3:** Factors associated with treatment outcome according to management strategy for group 1, group 2 and groups 1 and 2 combined

Factors associated with treatment outcome according to management strategy for group 1									
	Group 1			Group 1			Group 1		
	First line: collar			Second line: HBO			Summary: collar or HBO		
	Success	Intervention failure = progression to HBO		Success	Intervention failure = progression to surgery		Success	Treatment failure = progression to surgery	
All patients	9	10	Total = 19	4	6	Total = 10	13	6	Total = 19
Age (years) < 7 > 7	3 6	2 8	*p* = 0.44 (OR 0.5, 95% CI 0.033–6.13)	1 3	1 5	*p* = 0.67 (OR 0.6, 95% CI 0.01–62.65)	4 9	1 5	*p* = 0.47 (OR 0.45, 95% CI 0.01–6.79)
Gender Male Female	3 6	2 8	*p* = 0.44 (OR 0.5 95% CI 0.033–6.13)	2 2	0 6	*p* = 0.13 (OR 0, 95% CI 0–1.01)	5 8	0 6	*p* = 0.11 (OR 0, 95% CI 0–1.3)
Aetiology Traumatic Non-traumatic	3 6	5 5	*p* = 0.39 (OR 0.5, 95%CI 0.05–4.43)	3 1	2 4	*p* = 0.26 (OR 6, 95% CI 0.22–391.99)	6 7	2 4	*p* = 0.49 (OR 1.71, 95% CI 0.16–24.77)
Time to presentation < 1 month > 1 month	5 4	2 8	*p* = 0.13 (OR 0.2, 95% CI 0.01–2.11)	2 2	0 6	*p* = 0.13 (OR 0, 95% CI 0–1.03)	7 6	0 6	*p* = 0.03* (OR 0, 95% CI 0–0.69)
Factors associated with treatment outcome according to management strategy for group 2									
	Group 2 First line: HBO								
	Success			Failure = progression to surgery					
All patients	10			4			Total = 14		
Age (years) < 7 > 7	3 7			0 4			*p* = 0.33 (OR 0, 95% CI 0–3.06)		
Gender Male Female	7 3			1 3			*p* = 0.17 (OR 0.14, 95% CI 0.002–3.06)		
Aetiology Traumatic Non-traumatic	5 5			1 3			*p* = 0.41 (OR 3, 95% CI 0.15–188.44)		
Time to presentation < 1 month > 1 month	1 9			1 3			*p* = 0.51 (OR 3, 95% CI 0.03–261.12)		
Factors associated with treatment outcome according to management strategy for group 1 + 2 combined									
	All patients: group 1 + 2								
	Success			Failure = progression to surgery					
All patients	23			10			Total = 33		
Age (years) < 7 > 7	7 16			1 9			*p* = 0.21 (OR 3.94 95% CI 0.38–196.82)		
Gender Male Female	12 11			1 9			*p* = 0.02* (OR 9.8 95% CI 0.99–465.35)		
Aetiology Traumatic Non- traumatic	11 12			3 7			*p* = 0.29 (OR 0.47 95% CI 0.06–2.79)		
Time to presentation < 1 month > 1 month	8 15			1 9			*p* = 0.15 (OR 4.8 95% CI 0.47–236.21)		

## Surgically treated cases

**Fig. 3:**
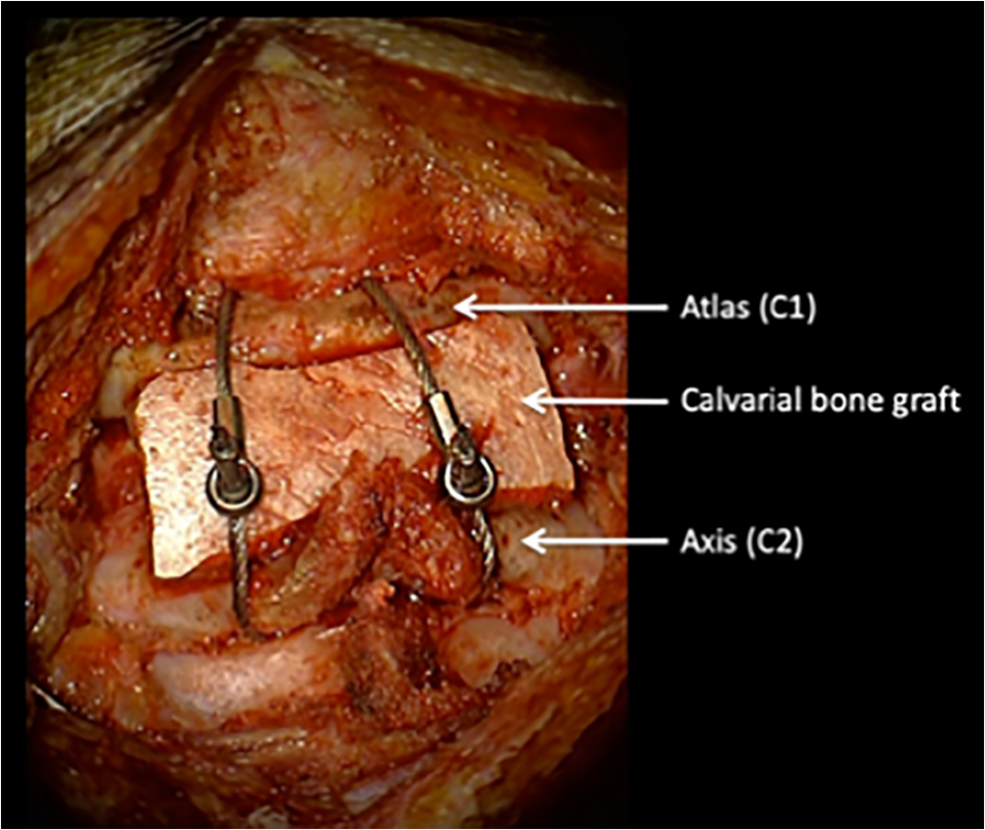
Gallie procedure. *Gallie procedure using sublaminar cables and calvarial bone graft in 8-year-old girl with relapsed AARF*

Ten patients underwent surgical fixation, all having failed prior treatment with closed reduction and HBO; 6 of these had also failed closed reduction and cervical collar. The surgical technique was tailored according to age and anatomical suitability. Four children underwent C1-2 lateral mass screw fixation; four underwent modified Gallie procedures using autologous calvarial bone graft secured with sublaminar cables (Atlas® Cable System, Medtronic) (Fig. 3). One patient underwent C0-C2 fixation with C1 decompression, as C1 lateral mass was unfavourable for instrumentation, and one patient was fused from C1 to C3. In 9 out of 10 surgical cases, complete reduction was achieved and maintained at follow-up. In one case, attempts at open reduction failed and *in situ* fixation was performed. More involved surgical techniques that have been described for AARF including transoral and far lateral approaches were not indicated for any of the patients in this series [18–20]. A summary of the demographics of the surgically treated patients is included in Table 4.

**Table 4:** Demographics of surgical treated patients. A summary table of the demographics of the surgically treated patients. Age refers to the age at presentation. Time to presentation refers to the time from the onset of a torticollis to first contact with the neurosurgical team

Patient	Age (years) and gender	Time to presentation	Mechanism	Treatment group	Surgery
1	8 female	62 days	Trauma	Group 1	C1-2 Sublaminar wires and calvarial graft
2	2.2 female	93 days	Idiopathic	Group 1	C1-3 fusion with autologous graft
3	11.7 female	77 days	Trauma	Group 1	C1-2 fixation with screws
4	11.1 female	31 days	Grisel’s syndrome	Group 1	C1-2 sublaminar wires and calvarial graft
5	7.5 male	70 days	Idiopathic	Group 1	C1-2 fixation with screws
6	10 female	248 days	Idiopathic	Group 1	C1-2 fixation with screws
7	7.8 female	62 days	Seizure	Group 2	C1-2 sublaminar wires and calvarial graft
8	7.6 female	365 days	Trauma	Group 2	C0-2 fixation with screws
9	9.1 female	62 days	Idiopathic	Group 2	C1-2 fixation with screws
10	5.3 female	7 days	Idiopathic	Group 2	C1-2 Sublaminar wires and calvarial graft

## Follow-up and complications of treatment

There were no adverse neurological or neurovascular events, no episodes of deep space infection and no instances of failed instrumentation. A superficial pin site infection occurred in a HBO patient assigned to group 2. This resolved with antibiotic management and pin re-positioning. No hospital admission or further sequalae was observed as a result of this complication. There were no other complications within the study period which includes a minimum of 12-month follow-up after completion of treatment for all patients.

## Discussion

Early diagnosis and effective initial reduction are key to successful management of AARF in childhood [10, 12, 14–17]. In many instances, this can be achieved through simple conservative measures, comprising analgesia, muscle relaxants and collars without MUA [21, 22]. However, where torticollis does not resolve promptly, escalation of treatment is essential as delay is a significant factor in poor long-term outcome [15, 19]. Whilst there is consensus within the neurosurgical community regarding the diagnostic criteria and imaging features of AARF, there are significant variations in treatment strategies. There are two particular points of controversy: what is the most effective way to achieve reduction of the C1-C2 deformity? How to maintain that reduction in order to reduce the risk of relapse?

**Fig. 4:**
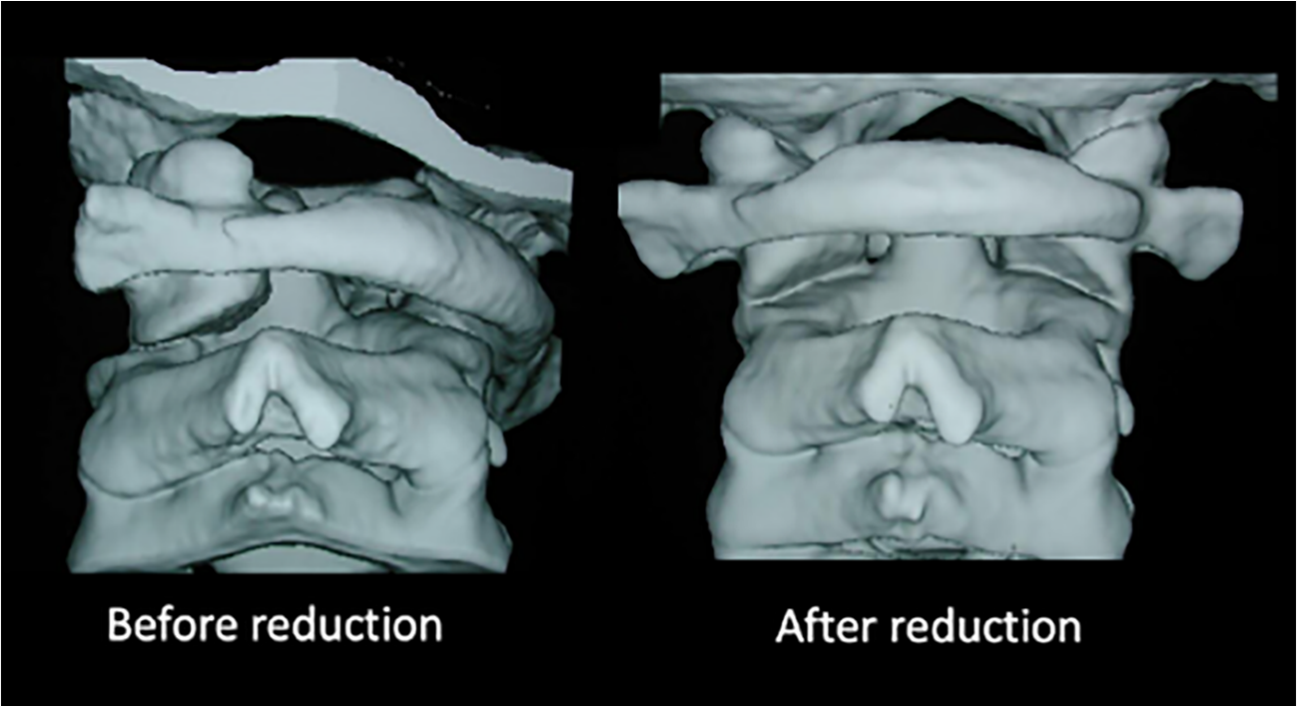
Rotational deformity of C1 in relation to C2 before and after reduction. *3D computerised tomographic reconstruction of the craniocervical junction demonstrating rotational deformity of C1 in relation to C2 before and after closed reduction under anaesthesia*

The two main options for achieving closed reduction are traction (Halter or skull traction) and MUA with radiographic screening [12, 21–24]. Whilst there are reports of successful outcomes using traction, patient compliance particularly in the very young will not infrequently preclude its use in the awake patient. Even where Halter traction is feasible, the duration of traction often necessitates prolonged hospitalisation. Durations of Halter traction reported in the literature include from 1 to 28 days [25] and as long as 6 weeks [26]. MUA permits immediate reduction of the deformity and avoids the need for confinement in bed or prolonged in patient stay [27]. However, amongst some neurosurgeons, there is concern about the risks of causing neurological or neurovascular injury through manipulation. All 33 cases in this series underwent MUA and we observed no instance of neurovascular injury or new neurological deficit. The technique, and safety of MUA for AARF, has been described previously [27]. In up to one third of patients, reduction occurs spontaneously on induction of anaesthesia but more usually, manual traction and manipulation of the deformity is required to realign C1 and C2 [27]. Radiologically confirmed reduction of AARF was achieved in all the cases in this series. Subsequent immobilisation in either a hard collar or HBO resulted in sustained resolution of torticollis and avoidance of surgery in 70% of cases in this series (23/33) (Fig. 4).

A CT scan was performed at end of treatment, after discontinuing hard collar use or removal of HBO body orthosis. This final scan was not routinely performed with left and right rotation. We reasoned that if there was no further clinical evidence of torticollis and the child had a good range of movement, then a neutral position CT scan showing normalised alignment was adequate evidence that AARF had been successfully treated and the extra irradiation incurred by dynamic imaging was not justified.

Children with late presentation, particularly those where there is evidence of new bone formation at the C1-C2 facet joints, may prove irreducible [13]. Whilst this does not usually pose any neurological risk to the child, there is no insignificant morbidity due to chronic pain, compensatory subaxial deformity and cosmetic consequences. Children with fixed deformities or those who failed attempted closed reduction are not included in this series as our aims in this study were to evaluate the treatment pathway and outcomes of those patients in whom closed reduction could be achieved.

Once reduction has been achieved, by whatever means, there is a high rate of early relapse if measures are not taken to immobilise the upper cervical spine [11, 15]. Relapse after primary treatment predicts an increased risk of the need for operative fusion in our series; this is consistent with previous reports [15].

Whilst instrumented fixation is the most definitive means of maintaining reduction, surgery is not without risk and there are justified concerns regarding the inevitable loss of range of motion that results from arthrodesis [12, 13, 18]. In this paper, we have demonstrated that up to 70% of children with AARF can be safely and effectively successfully treated by MUA and immobilisation alone and can avoid the need for internal fixation.

What is unclear is whether cervical collar or HBO body orthosis is more effective in maintaining reduction following MUA and thus avoiding the need for surgery? A limitation of this study is that over the time period under consideration, the policy regarding immobilisation has changed. Moreover, children were not randomly assigned to cervical collar or HBO and so any interpretation of the efficacy of one treatment strategy over the other has to be made with caution. The treating surgeon decided the type of post-MUA immobilisation on a case-by-case basis; this decision was not formalised or directed by any specific guideline. HBO body orthoses provide greater immobilisation than rigid cervical collars; however, they are unpopular with children and families and morbidity, such as pin loosening, and pin site infection is reported in up to 40% case in some series [28]. In this series, HBO as part of first-line management was effective in avoiding open surgery in 71% of cases, and effective in 58% of cases overall. Cervical collar was effective in avoiding surgery in almost half of cases (47%) when used as first-line treatment. Although not statistically significant, there was a tendency for MUA and collar to be more efficacious when used in cases with shorter history. On the basis of our findings, it is not possible to offer categorical recommendations for the use of HBO over collar. However, it seems that for children with short symptom duration, who can be easily reduced by MUA, a period of immobilisation in a hard collar supplemented by close clinical surveillance is a reasonable option. Children and their parents need to be warned of the risks of relapse and the potential need to escalate treatment.

The importance of early diagnosis and treatment has been highlighted in other paediatric series. Pang and Li found that no acute presentations required fusion, whereas chronic cases required fusion in up to half of cases [11]. Similarly, Beier et al. found that out of 29 patients who presented within 1 month, none required fusion, compared with 3 out of 5 who presented subacutely between 1 and 3 months [16]. In the current study, we observed that of the children treated within 1 month of symptom onset, 89% were successfully managed without recourse to surgery, compared with 62% whose history was greater than 1 month (*p* = 0.13). Nine of the ten patients requiring surgery presented more than 1 month from the onset of symptoms.

The overall success rate for the group 1 strategy was 47% (9/19). This is lower than the 71% (10/14) rate for those initially treated with MUA and HBO (group 2). When used as a first-line treatment (group 2), the success of a HBO was 71% (10/14) compared with when used as a second line (group 1) where success was only 40% (4/10). This raises the possibility that using HBO as a second line may be detrimental as such a policy incurs an additional time delay to definitive treatment, resulting in a greater surgical conversion rate. However, this did not reach significance.

No significant association was found between the cause of AARF or the age of patients and the overall success of individual strategies or overall non-surgical management. This is in keeping with previous findings of Pang et al. [11, 15]. Although there were more females in this study, the published literature suggests that there is no clear gender predisposition to AARF.

However, it was found that overall success rates for non-surgical management (groups 1 and 2 combined) were significantly higher for male patients as compared with female patients (79% vs 63%). The trend was sustained when the groups were analysed individually but did not reach a level that could be considered significant. We did not find any definitive assessment of outcome differences related to gender. In contrast to non-operative approaches, surgical intervention has a high rate of success rate (100% in our series). However, due to its invasiveness and associated risks, we generally reserve this option if the AARF fails to resolve with non-operative care or there is a clear indication for surgery such as an associated traumatic spinal injury mandating surgical intervention.

A number of factors influenced the decision to use a collar or HBO after MUA; these included perceived risk of recurrence, presumed patient compliance and parental preference. This allocation bias represents one of the main limitations of this study. Furthermore, given the relative rarity of this condition, the numbers are small precluding accurate subgroup analysis or comparison. Nonetheless, it is hoped that this experience will add to the limited literature on this topic and help inform future guidelines.

## Conclusion

Early diagnosis of AARF is essential as delay in treatment can preclude a successful reduction. After a brief period of conservative treatment with analgesia, muscle relaxants and physiotherapy, we recommend prompt reduction followed by immobilisation as a first-line strategy. MUA is a safe, immediate and effective means of reducing the rotatory deformity in cases of AARF and avoids prolonged hospital stay associated with traction. The efficacy of MUA followed by HBO immobilisation is greater when used as an initial treatment strategy than when used after relapse following MUA with collar. Children who present late have a higher relapse rate after immobilisation by either collar or HBO and are more likely to require internal fixation. The majority of children (70%) can be successfully treated without recourse to open surgery and this data supports a policy of escalating treatment in an attempt to avoid the risks and long-term implications of instrumented fixation.
